# Effect of fentanyl and remifentanil on neuron damage and oxidative stress during induction neurotoxicity

**DOI:** 10.1111/jcmm.18118

**Published:** 2024-02-08

**Authors:** Ali Taghizadehghalehjoughi, Muhammet Emin Naldan, Yesim Yeni, Sidika Genc, Ahmet Hacimuftuoglu, Mesut Isik, Adem Necip, İsmail Bolat, Serkan Yildirim, Sukru Beydemir, Mahmut Baykan

**Affiliations:** ^1^ Department of Medical Pharmacology, Faculty of Medicine Bilecik Seyh Edebali University Bilecik Turkey; ^2^ Department of Anesthesiology and Reanimation University of Health Sciences, Hospital of City Erzurum Turkey; ^3^ Department of Medical Pharmacology, Faculty of Medicine Ataturk University Erzurum Turkey; ^4^ Department of Bioengineering, Faculty of Engineering Bilecik Seyh Edebali University Bilecik Turkey; ^5^ Department of Pharmacy Services, Vocational School of Health Services Harran University Sanlıurfa Turkey; ^6^ Department of Pathology, Faculty of Veterinary Medicine Ataturk University Erzurum Turkey; ^7^ Department of Biochemistry, Faculty of Pharmacy Anadolu University Eskisehir Turkey; ^8^ The Rectorate of Bilecik Seyh Edebali University Bilecik Turkey; ^9^ Department of Microbiology, Faculty of Medicine Bilecik Seyh Edebali University Bilecik Turkey

**Keywords:** cholinergic system, fentanyl, neuron, neurotoxicity, remifentanil

## Abstract

Opioids can be used for medical and non‐medical purposes. Chronic pain such as cancer, as well as the frequent use of such drugs in places such as operating rooms and intensive care units, and in non‐medical areas like drug abuse the effects and side effects of these drugs need to be examined in more detail. For this purpose, the effects of fentanyl and remifentanil drugs on neuroinflammation, oxidative stress and cholinesterase metabolism were investigated. Neuron cells (CRL‐10742) were used for the evaluation of the toxicity of fentanyl and remifentanil. MTT, PON1 activity and total thiol levels for its effect on oxidative stress, AChE and BChE activities for its effect on the cholinergic system, and TNF, IL‐8 and IL‐10 gene levels for its neuroinflammation effect were determined. The highest neurotoxic dose of fentanyl and remifentanil was determined as 10 μg/mL. It was observed that the rate of neuron cells in this dose has decreased by up to 61.80% and 56.89%, respectively. The IL‐8 gene expression level in both opioids was down‐regulated while IL 10 gene level was up‐regulated in a dose‐dependent manner compared to the control. In our results, the TNF gene expression level differs between the two opioids. In the fentanyl group, it was seen to be up‐regulated in a dose‐dependent manner compared to the control. Fentanyl and remifentanil showed an inhibitory effect against PON1, while remifentanil showed an increase in total thiol levels. PON1, BChE and total thiol activities showed similarity with MTT.

## 1 INTRODUCTION

Deaths, due to opioids, are on the rise worldwide. Most of the overdose deaths were from opioids, most of them with fentanyl.^1^ Prescriptions of opioids have decreased considerably today, but deaths have nevertheless increased. This indicates that the illicit use of fentanyl is increasing.^2^ There have been reports describing an increase in opioid‐related disability, as well as an increase in opioid‐related overdose deaths. Exposure to opioids induces volumetric changes in the brain that can cause changes in various brain regions, including areas related to cognition such as the hippocampus.^3^ Opioids are known to induce neuronal cell death and impair neurogenesis.^4^, ^5^, ^6^, ^7^ In addition, it has been observed that loss of impulse control occurs in opioid addicts by affecting the frontal cortex.^8^

In addition, the impaired neuron regeneration process and mitochondrial dysfunction are strongly related.^9^ It is known that mitochondria are essential for the proper maintenance of cellular functions, apoptosis and cell death. Impaired mitochondrial function is observed in various neurodegenerative diseases such as Parkinson's and Alzheimer's diseases,^10^ and possibly mitochondrial deficiencies are also sources of opioid‐induced cognitive impairment.

In vitro studies show that opioids inhibit mitochondrial activity in human beings. It has been thought that opioids reduce mitochondrial respiration in primary neuronal cell cultures^6^, ^11^, ^12^ and hepatic cells,^13^ and inhibiting mitochondrial ATP synthesis in human brain cells induces cellular necrosis.^7^ However, studies describing the effects of opioids on mitochondrial morphology are limited. Given the accumulating evidence that impaired mitochondrial function inhibits cellular function and that opioids can induce neuronal cells, it is necessary to examine the effects of opioids on a cellular basis. In addition, the deterioration of cognitive functions such as psychomotor functionality, information processing, attention, problem‐solving and memory impairs the social functions of the patients.

Both the increased use of synthetic opioids in non‐medical ways and the increase in the use of opioids due to chronic pain such as cancer, as well as the frequent use of such drugs in places such as operating rooms and intensive care units: the effects and side effects of these drugs need to be examined in more detail.

In this study, we focused on the most commonly used analgesics fentanyl and remifentanil. This has led us to focus on neurodegenerative and oxidative damages that may occur in neuron cells with the increase in the use of drugs. Inflammatory responses play an important role in the pathophysiology of various neurodegenerative diseases. In this study, neuroinflammation levels were determined by examining the effects of drugs on the synthesis and release of TNF, IL‐8 and IL‐10, which are important inflammatory biomarkers. Moreover, the effects of these drugs on acetylcholinesterase (AChE) and butyrylcholinesterase (BChE) activities in the cholinergic system associated with Alzheimer's disease, which is one of the neurodegenerative diseases, and on Paraoxonase 1 (PON1) activity and total thiol level, which play a role in the cellular antioxidant defence system, were determined.

## 2 MATERIALS AND METHODS

### 2.1 Chemicals and reagents

Fentanyl and remifentanil were purchased from Belgium (Genval). Neurobasal medium (NBM), MTT, fetal bovine serum (FBS), antibiotic antimitotic solution and trypsin–EDTA were obtained from Sigma‐Aldrich (St. Louis, MO, USA). Total antioxidant capacity (TAC) and total oxidant status (TOS) were obtained from Turkey, Rel Assay Diagnostics.

### 2.2 Primer cell cultures

In the study, Sprague Dawley (a newborn) rat that had not completed 24 h was used to obtain cortex neurons. Briefly, after the rats were decapitated quickly, the removed cortices were transferred to 5 mL of phosphate buffer solution (PBS), and macro‐fragmentation was performed with the help of a scalpel and then micro‐fragmentation was performed with Trypsin (0.02%). The cells were then centrifuged at 200*g* for 5 min. Cells sinking to the bottom are cellular medium, 88% NBM (Neurobasal medium, Gibco, USA), 10% FBS (Fetal bovine solution, Gibco, USA), 2% B‐27% (Supplement, Thermo Fisher, Germany), 0.1% antibiotic (Penicillin–Streptomycin) and amphotericin B (Thermo Fisher, Germany) were added. For 10 days, the cells were incubated at 5% CO_2_ and 37°C, changing the medium every 3 days.^14^

**FIGURE 1:**
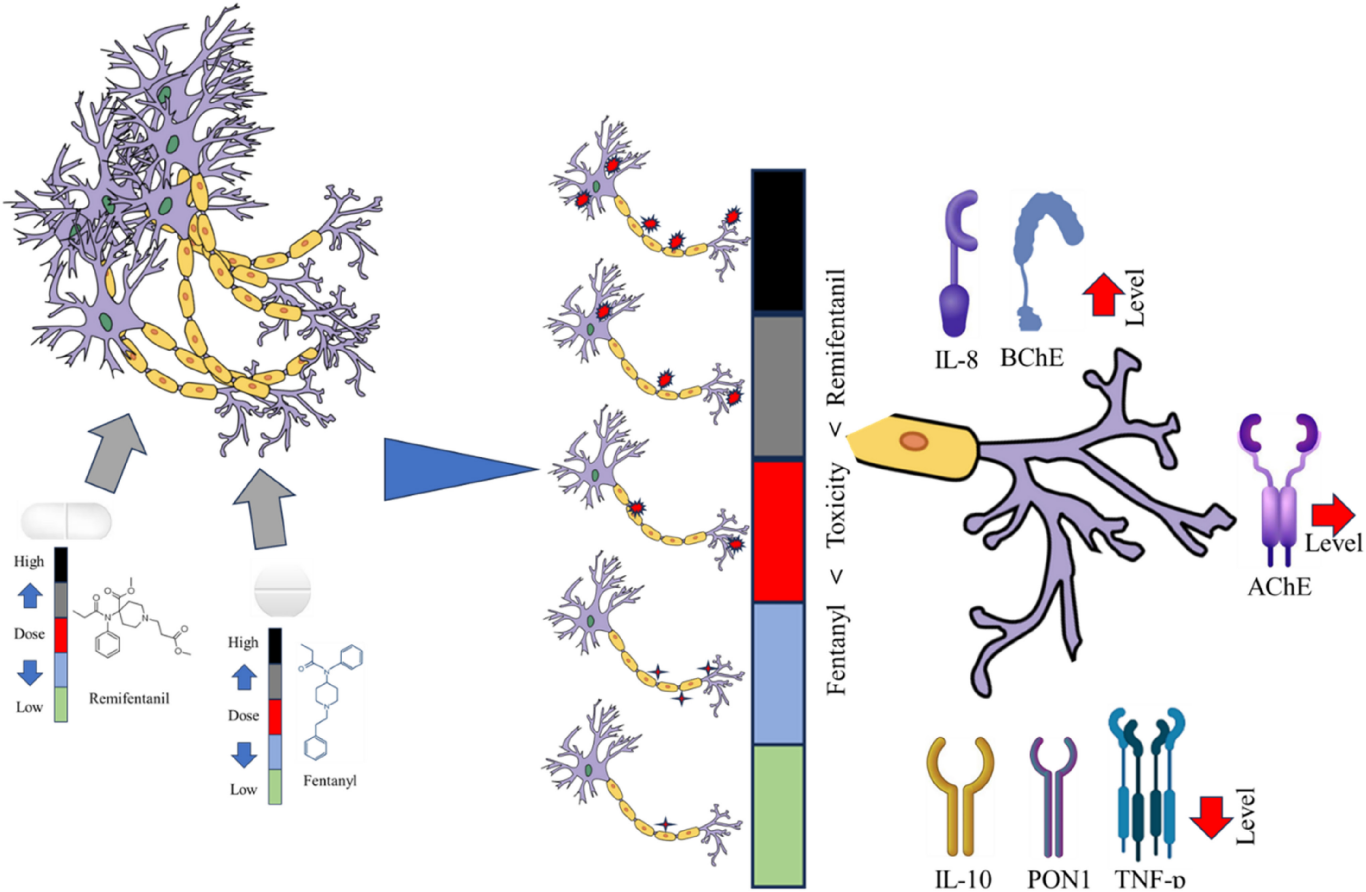
Neuron culture was established. Fentanyl and remifentanil were administrated. Different markers were evaluated.

### 2.3 MTT assay analyses

According to the kit protocol, an MTT assay was applied. For this purpose, 10 μL MTT reagent was added to each well and incubated for 4 h. Then, dimethyl sulfoxide (100 μL) was added.^15^ The optical density was evaluated at 570 nm, and the cell viability (%) was calculated using the formula:

$$\text{Viability}\;\%\;\text{ratio}=\frac{\text{sample absorbance value}}{\text{control group absorbance value}}\times 100$$

### 2.4 Real‐time PCR analysis

#### 2.4.1 Obtaining genetic material

The medium of the 6‐well plates was removed, and after washing with PBS, 0.2 mL of Trypsin/EDTA was added to each well, and time was given for 5 min to remove the cells. FBS was added at a ratio of 1:1, and the cells floating in the flask solution were taken into a sterile tube; then, for 5 min, it was centrifuged at 1200 rpm. A cell pellet was taken for RNA isolation Figure 1.

#### 2.4.2 RNA isolation

RNA isolation was done with the Roche isolation kit. The cell pellets were centrifuged at 12,000 *g* for 15 min at 4°C. The colourless liquid and ethanol alcohol were added at a ratio of 1:1 and vortexed for 5 s. The sample (700 μL) was placed in the collection tube and centrifuged at 8000 *g* for 15 s at room temperature with the lid closed. In the RNeasy column, 700 μL of Wash buffer 1 solution was added and centrifuged. Then, 10 μL DNase and 90 μL DNase Incubation Buffer and wait for 15 min. After then, wash buffer 2 was added (700 μL) and centrifuged. All centrifuged was done at 8000 *g* for 15 s. In this stage, a new 1.5 mL tube was placed on the RNeasy column and 50–100 μL of elution buffer was added and centrifuged at 8000 *g* for 1 min.

#### 2.4.3 cDNA synthesis

Two microliter of genomic DNA, for cDNA synthesis, wipe out buffer 7× solution plus 1 μg of RNA and 14 μL of RNase‐free water were prepared and kept on ice for 2 min at 42°C. Then, 1 μL of reverse transcription master mix, 5× 4 μL of Quantrscript RT buffer, 1 μL of RT primer mix, 14 μL of RNA and a total of 20 μL were added. The cDNA formation was carried out for 15 min by heating at 42° and 95° for 3 min, and the resulting cDNAs will be lifted to −20°.

#### 2.4.4 Gene expression

At this stage, 1 μL of probe, 3 μL of cDNA, 3 μL master mix and 13 μL of distilled water were added to each strip and the final volume was adjusted to 20 μL. After waiting for 600 s at 95°C, a total of 45 cycles were made for 10 s at 95° and 30 s at 60°.

#### 2.4.5 Measurement of Paraoxonase 1 (PON1) activity

To evaluate PON1 activity, it was kept at 25°C with paraoxone (diethyl p‐nitrophenyl phosphate; 1 mM) in 50 mM glycine/NaOH (pH 10.5) containing 1 mM CaCl_2_. The paraoxonase enzyme analysis was based on the estimation of p‐nitrophenol at 412 nm. The molar extinction coefficient of p‐nitrophenol (€ = 18.290 M^−1^ cm^−1^ at pH 10.5) was used to calculate PON1 activity.^16^ One enzyme unit was defined as the amount of enzyme that catalyses the hydrolysis of 1 mmol of substrate at 25°C. Evaluations were made using spectrophotometric (Chebios UV–VIS) measurements.^17^

#### 2.4.6 The cholinesterase activity assay

After 24‐h administration with fentanyl and remifentanil, cells were removed from the well by treatment with trypsin. The cells were sonicated on ice for 20 min and then centrifuged at 100,000 *g* at 4°C, and the supernatant was collected. Enzyme activity was determined using a colorimetric assay employing acetylthiocholine iodide (ASChI) for AChE and butyrylthiocholine iodide (BTChI) for BChE as substrate.^18^, ^19^, ^20^ The reaction was started with the addition of substrate (ASChI/BTChI), after a 3‐min equilibration. The substrate's hydrolysis was determined by monitoring the change in absorbance at 412 nm.

#### 2.4.7 Measurement of total thiol amount

Total thiols were evaluated according to the Sedlak and Raymond method.^21^ Cell samples (0.1 mL) were mixed with 1.5 mL of 0.2 M Tris buffer (pH 8.2) and 0.1 mL of 0.01 M DTNB. The mixture was made up to 10 mL with methanol and throughout 30 min and incubated subsequently; then, it was centrifuged 15 min at 3000 rpm and assayed at 412 nm. To calculate total thiols, standard graphs were used.

#### 2.4.8 Immunohistochemical staining

Apoptotic cell death and morphological changes were performed using DAPI and 8‐OHDG fluorescent nuclear dye. DAPI and 8‐OHDG stain cells undergoing apoptosis characterized by chromatin condensation and nuclear fragmentation. Neuron cells were fixed in paraformaldehyde for 48 h at 4°C. After 48 h, cells were stained with DAPI and 8‐OHDG at room temperature, placed on slides and observed under a fluorescence microscope (magnification, 200×) to detect features of apoptosis.

#### 2.4.9 Statistical analysis

Statistical analysis was evaluated using one‐way analysis of variance (ANOVA) and Tukey's HSD test on SPSS 21.0 software.

## 3 RESULTS

### 3.1 MTT results

To research the cytotoxic effects of fentanyl and remifentanil on the neuron cell line, an MTT assay, a colorimetric method, was performed. Accordingly, after 24 h of exposure to fentanyl and remifentanil were encountered, the death of cells was equivalent to or greater than the control group.

The cell viability rate in the control group was accepted as 100. Other groups were compared with the control group. The highest neurotoxic dose of fentanyl and remifentanil was determined as 10 μg/mL. It is seen that the rate of neuron cells in this dose has decreased by up to 61.80% and 56.89%, respectively. According to our results, it has been determined that all doses of fentanyl and remifentanil are neurotoxic, but the most effective results are in the range of 1–10 μg/mL. According to the results of MTT analysis, the lowest effective dose was determined as 0.001 μg/mL compared to other doses and control.

**FIGURE 2:**
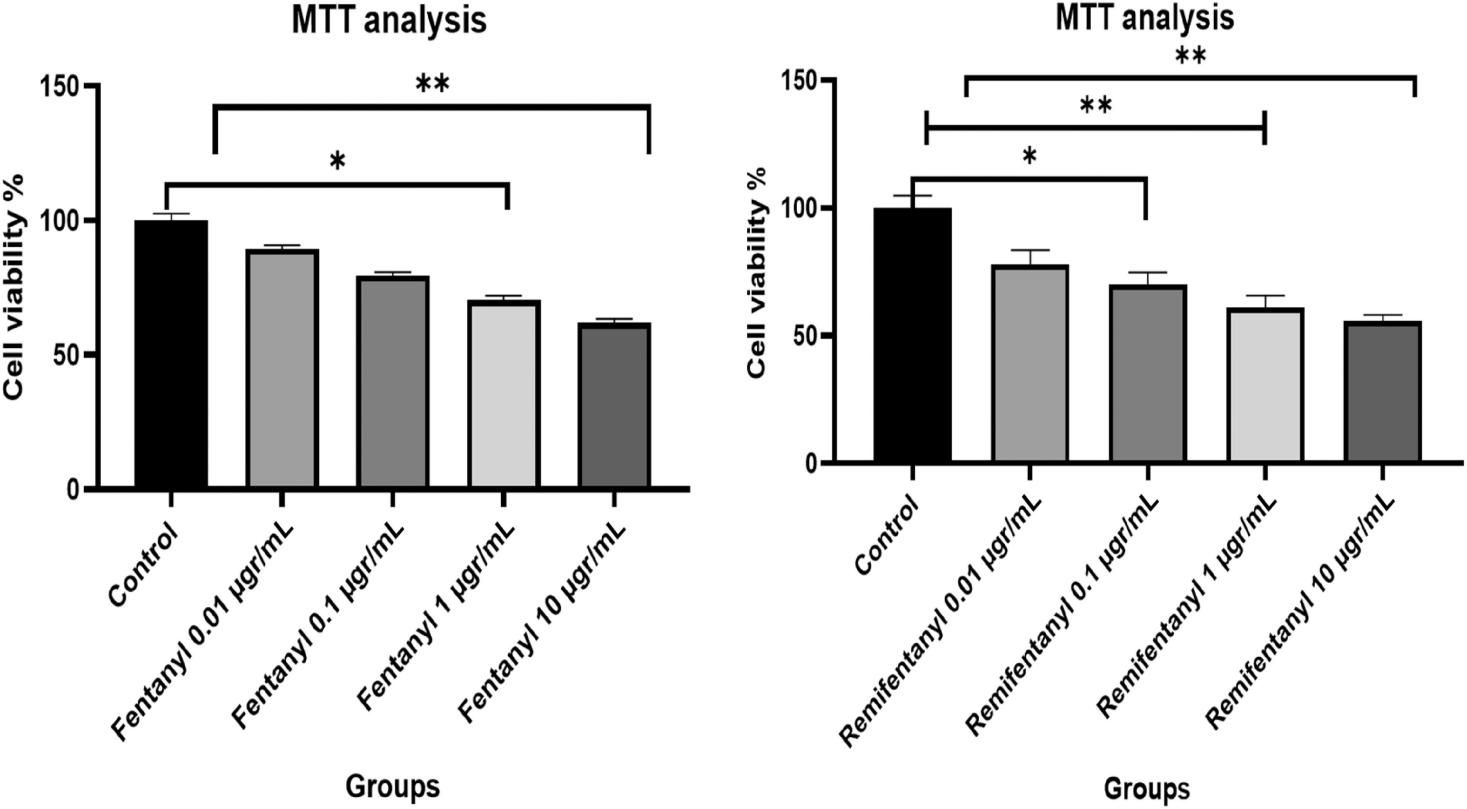
Reduction of neuron viability (MTT) after treatment with (A) fentanyl (B) remifentanil. Neuron cells were cultured in 96‐well plates treated with fentanyl and remifentanil (0.01, 0.1, 1, 10 μg/mL). The results represent the average of three separate experiments. One‐way ANOVA *p < 0.05; **p < 0.01.

Fentanyl and remifentanil have been found to reduce the dose‐dependent viability of the neuron cells, and the inhibition concentration results (IC_50_) are approximately 8.22 and 11.37 μg/mL (Figure 2).

### 3.2 RT‐PCR results

To further understand the molecular mechanisms involved in opioid (both fentanyl and remifentanil) induced inhibition of cytokine release, we studied the effect of opioids on the levels of mRNA for IL‐8, TNF and IL‐10 using real‐time RT‐PCR. RT‐PCR analysis is shown in Figure 2, and the control group value was accepted as 1.

It was observed that the IL‐8 gene expression level in both opioids was down‐regulated in a dose‐dependent manner compared to the control. However, the maximum decrease was observed at the dose of 10 μg/mL. While it was observed as 0.086 at a dose of 10 μg/mL fentanyl, this value was found to be 0.341363 at a dose of 10 μg/mL for remifentanil.

In our results, the TNF gene expression level differs between the two opioids. In the fentanyl group, it was found to be up‐regulated in a dose‐dependent manner compared to the control. The greatest increase was observed at the dose of 10 μg/mL (approximately 2.38‐fold). In the remifentanil group, on the other hand, downregulation was observed compared to the control group depending on the dose, and the highest decrease was found at the dose of 10 μg/mL (0.60) (*p* < 0.01).

**FIGURE 3:**
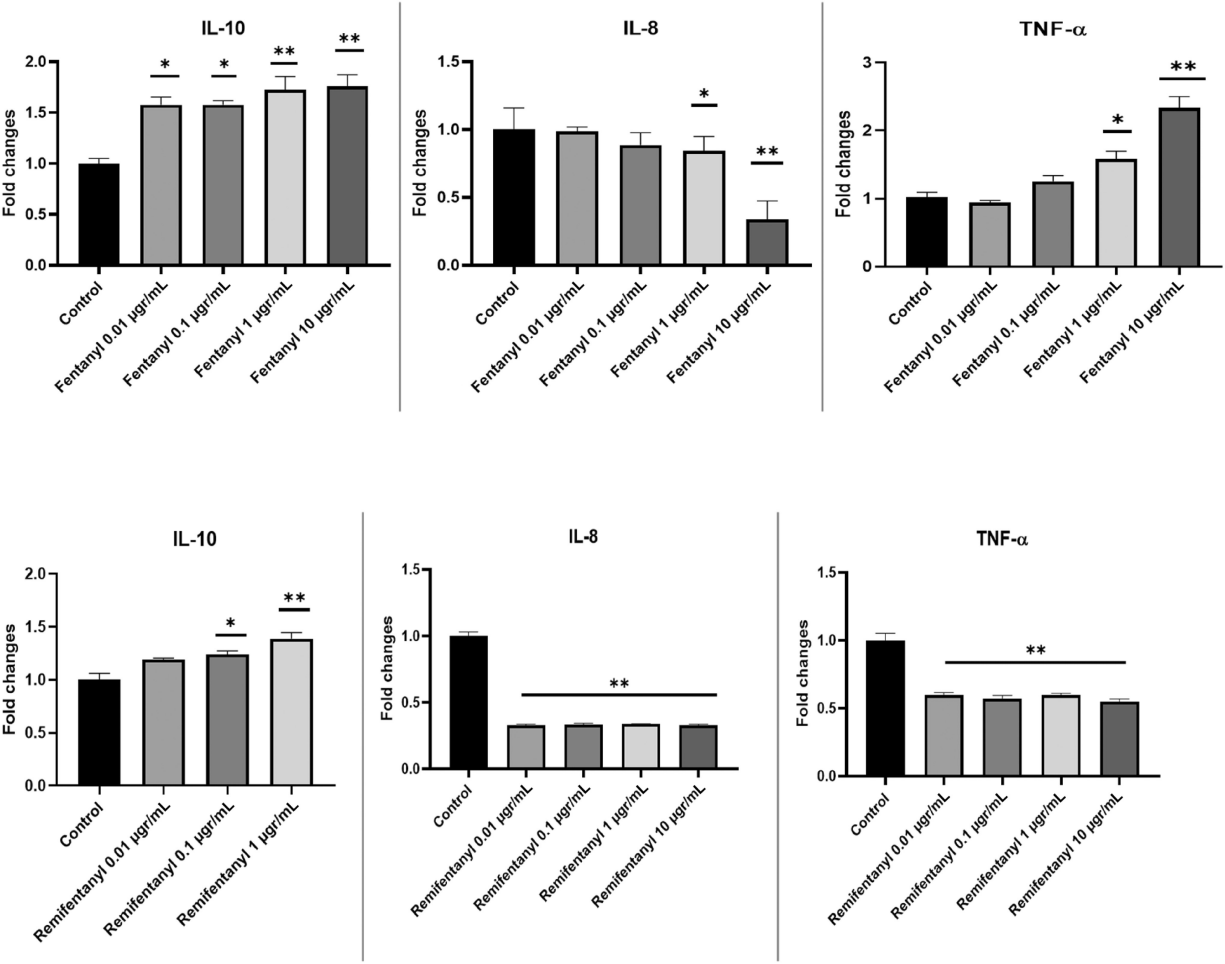
RNA expression levels of IL‐10, IL‐8 and TNF‐α in the neuron cell of control and experimental groups. (*p < 0.05; **p < 0.001; by *t*‐test; GraphPad Prism 7.0). (The β‐actin reference gene was used to standardize the mRNA levels.).

Similarly, IL 10 gene level was observed to be dose‐dependently up‐regulated in both opioid groups compared to the control group (*p* < 0.05). In the highest concentration, IL‐10 level was found to be 1.65 at fentanyl 10 μg/mL and 1.68 at remifentanil 10 μg/mL (Figure 3).

### 3.3 The results of Paraoxonase 1 (PON1) activity

**FIGURE 4:**
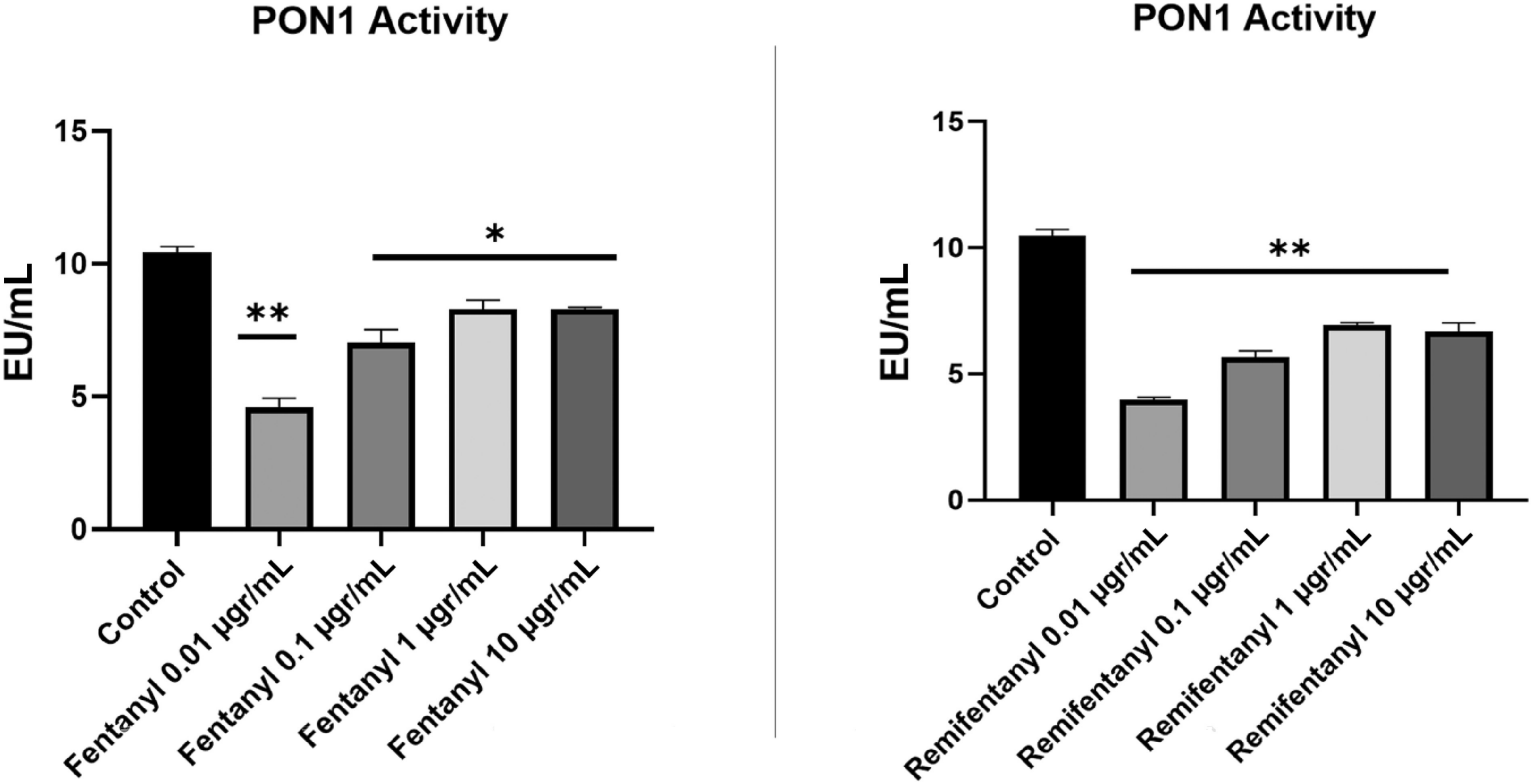
The PON1 activity results of fentanyl and remifentanil (0.01, 0.1, 1, 10 μg/mL). The results represent the average of three separate experiments. One‐way ANOVA *p < 0.05; **p < 0.01.

In the study, it was determined that fentanyl and remifentanil drugs applied at different doses caused inhibition of PON 1 activity in neuron cell culture when compared to controls. It was found that in the fentanyl group, it decreased to 4.92 at most. In the remifentanil group, while this value was 4.10, it was observed that there was a decrease independent of the concentration. The remifentanil group has a higher inhibitory effect on PON 1 than the other drug (Figure 4).

### 3.4 The results of the cholinesterase activity assay

**FIGURE 5:**
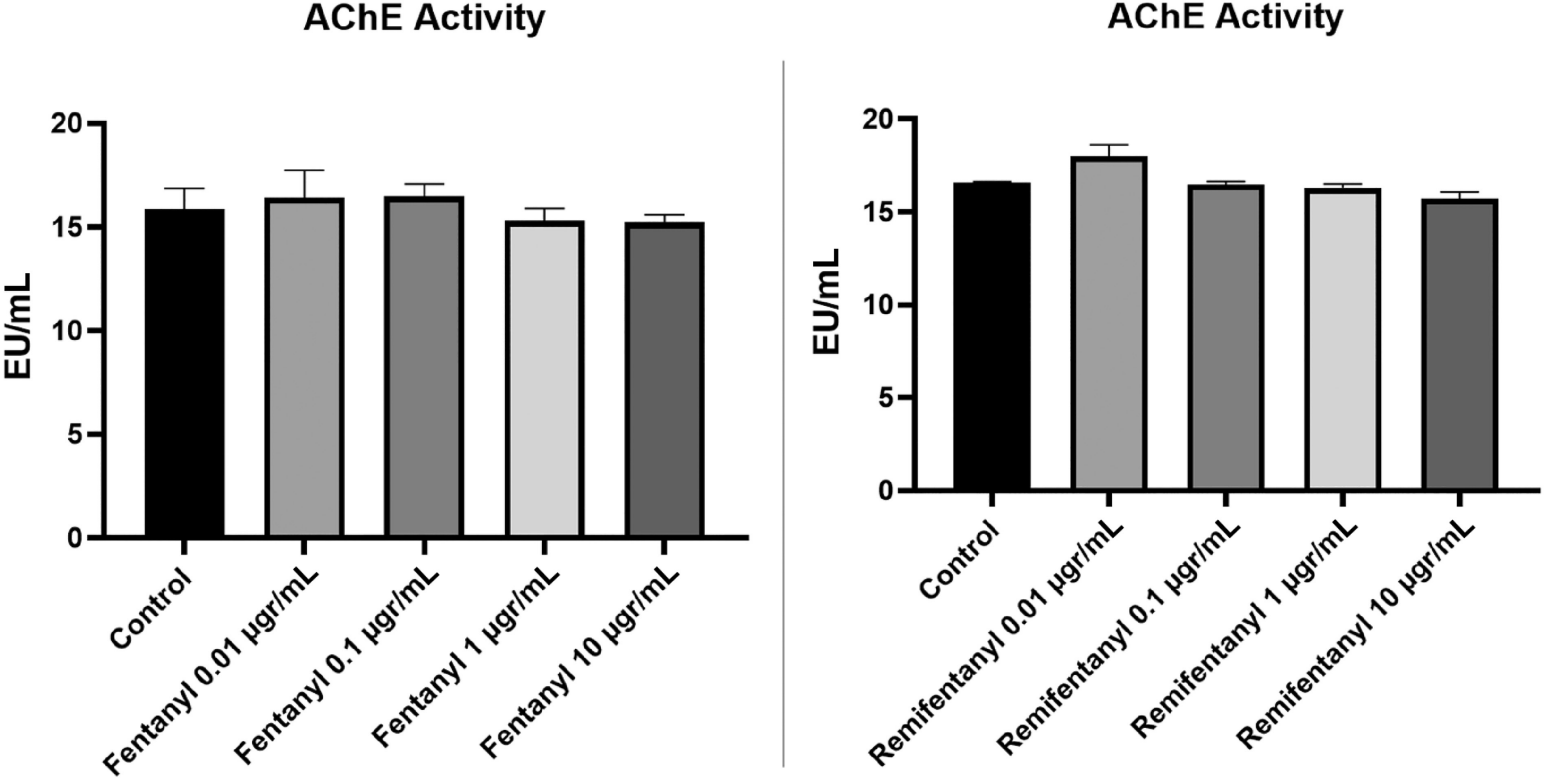
The AChE activity results of fentanyl and remifentanil (0.01, 0.1, 1, 10 μg/mL). The results represent the average of three separate experiments. One‐way ANOVA.

In this study, it was observed that fentanyl and remifentanil drugs applied at different doses to neuron cell culture did not show any effect against the AChE enzyme when compared to the control group but showed an inhibitory effect against BChE (Figure 5).

The AChE value of the control group was found to be 16.49. This value decreased to a maximum of 15.35 and 15.95 at the highest dose of both drugs. The results obtained were not statistically significant (*p* > 0.05).

**FIGURE 6:**
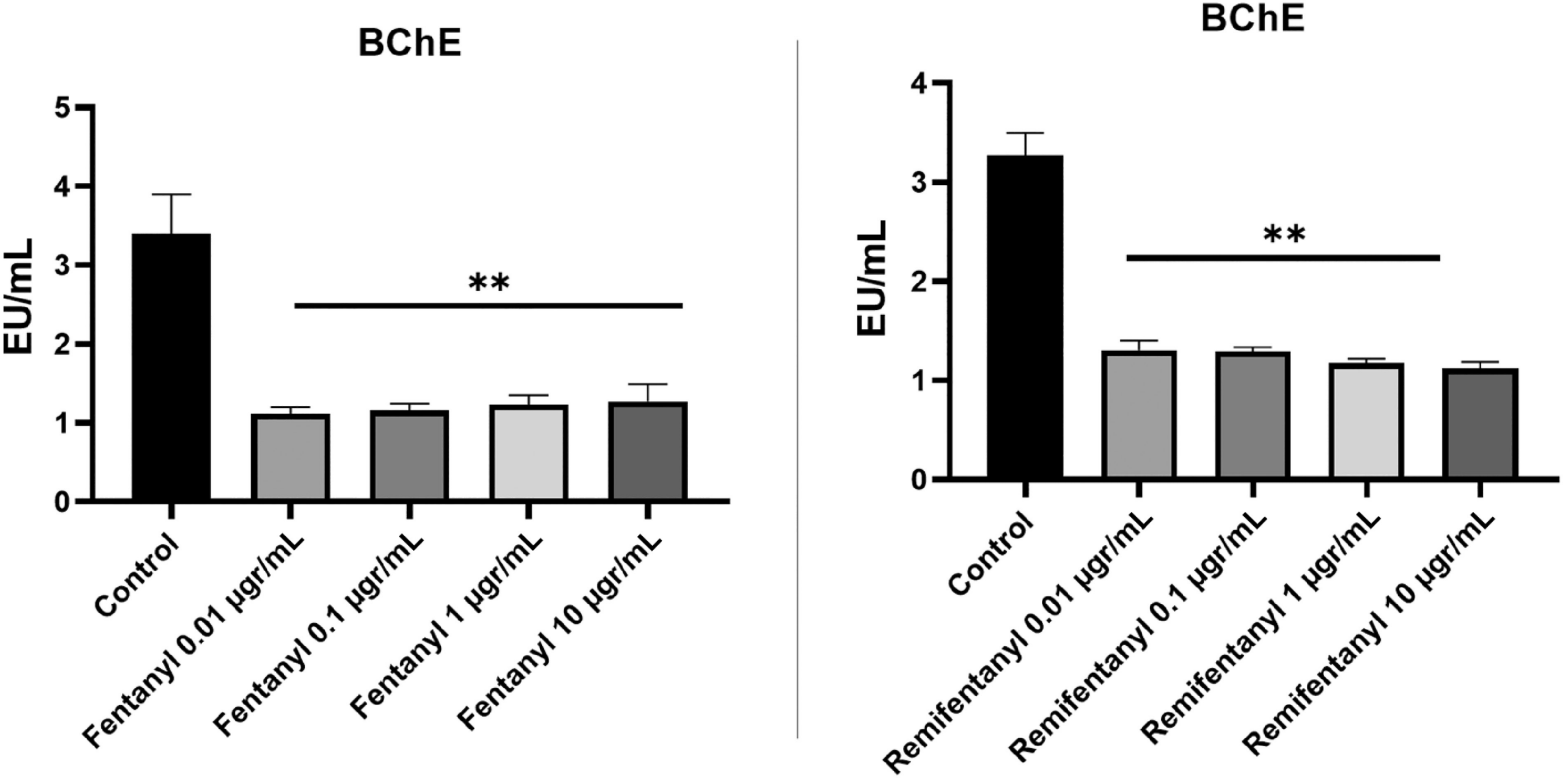
The BChE activity results of fentanyl and remifentanil (0.01, 0.1, 1, 10 μg/mL). The results represent the average of three separate experiments. One‐way ANOVA. **p < 0.01.

When the BChE activity was examined, the control group was found as 3.01. This value decreased to 1.19 in the fentanyl group and 1.14 in the remifentanil group. The data obtained were independent of the concentration, and the results of all groups were found to be statistically significant (*p* < 0.01) (Figure 6).

### 3.5 Results of total thiol amount

**FIGURE 7:**
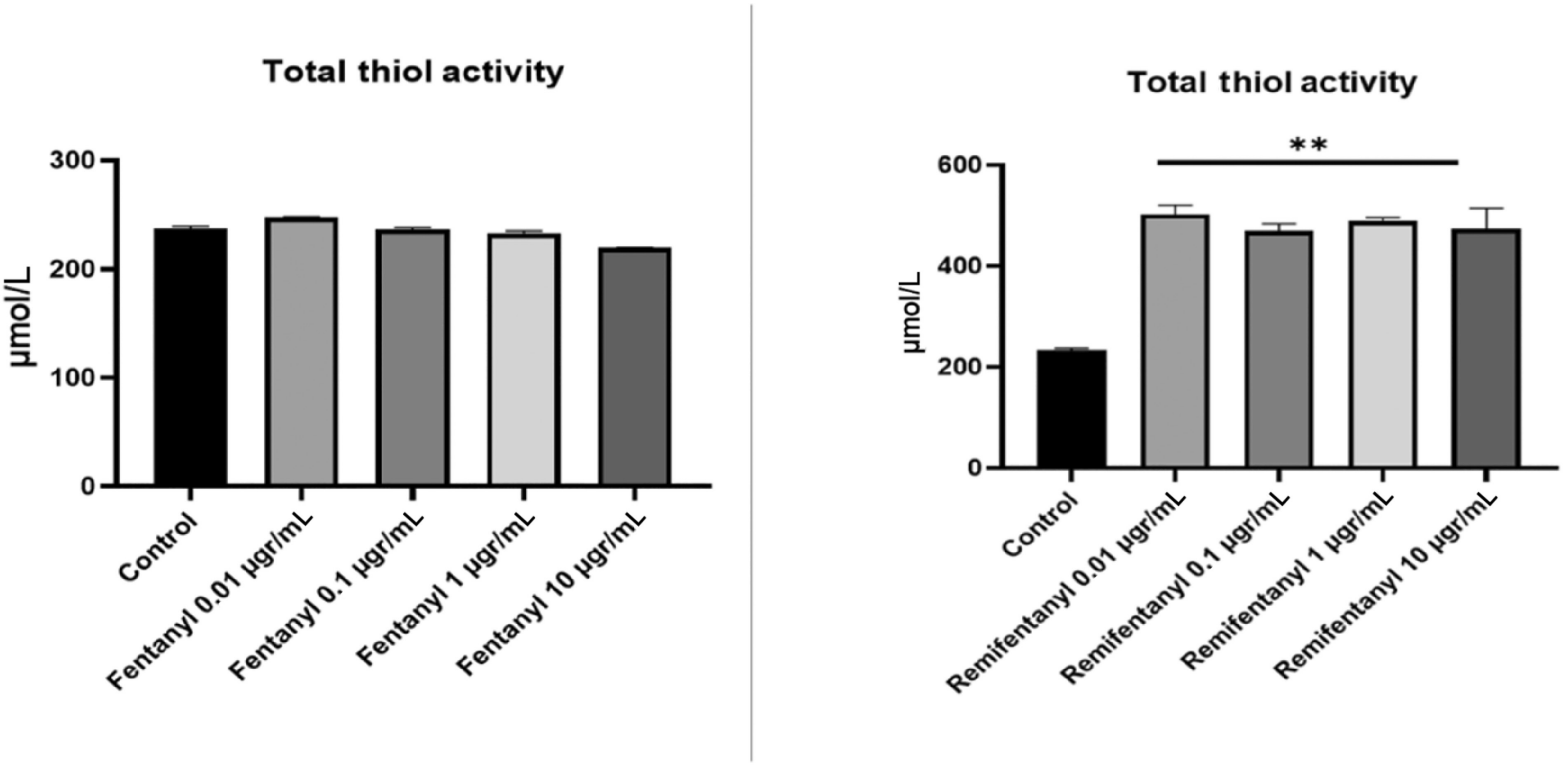
The total thiol activity results of fentanyl and remifentanil (0.01, 0.1, 1, 10 μg/mL). The results represent the average of three separate experiments. One‐way ANOVA. **p < 0.01.

In the study, when compared to controls, remifentanil applied at different doses increased the total thiol level in neuron cell culture, but no change was observed in the fentanyl group. The total thiol level, which was 237.27 in the control group, was found to be 219.81 in the highest concentration in the fentanyl group. The results of this group were not statistically significant (*p* > 0.05). In the remifentanil group, this value increased independently of the concentration, and the highest was found to be 523.68 (*p* < 0.01). One of the main reasons for this increase is the increase in the amount of thiol in the cell by activating the enzymatic (CAT, GPx and GR) and non‐enzymatic antioxidant defence system against oxidative stress caused by neurotoxicity caused by drugs (Figure 7).

### 3.6 Results of immunohistochemical staining

**FIGURE 8:**
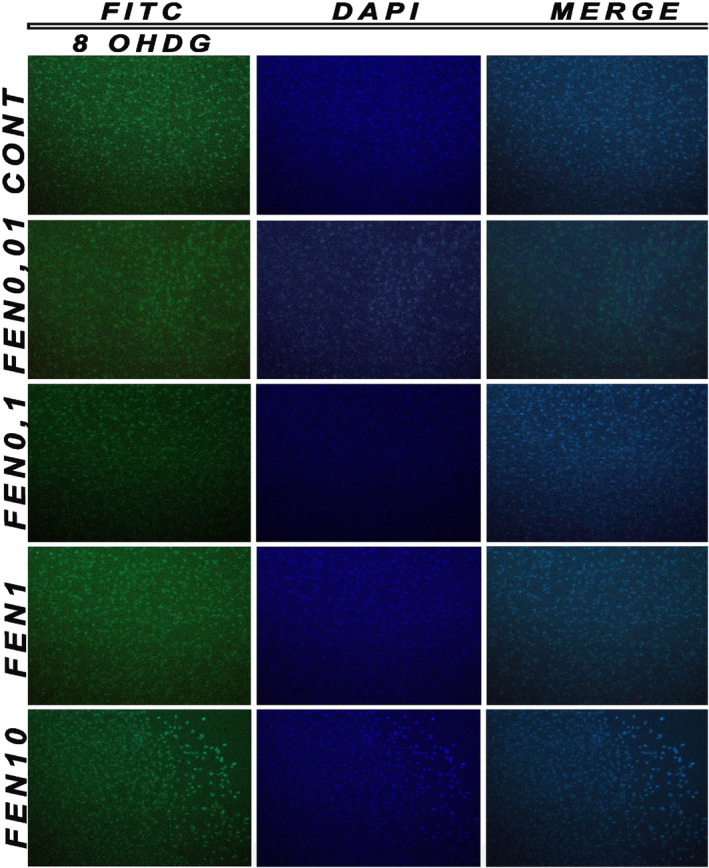
8‐OHdG and DAPI images of fentanyl (0.01, 0.1, 1, 10 μg/mL). The results represent the average of three separate experiments.

**FIGURE 9:**
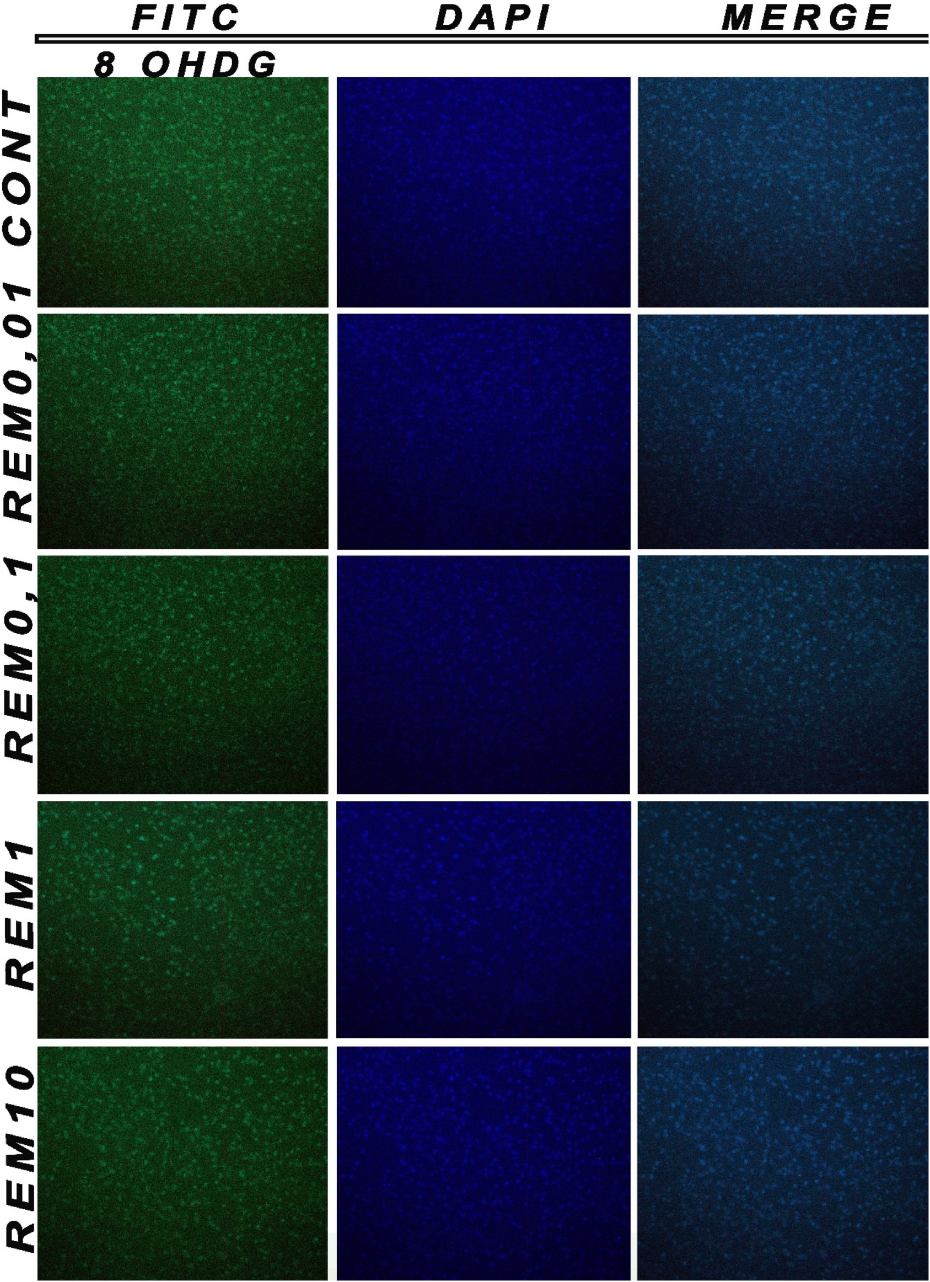
8‐OHdG and DAPI images of remifentanil (0.01, 0.1, 1, 10 μg/mL). The results represent the average of three separate experiments.

To determine whether the decreased cell viability observed in fentanyl and remifentanil cells was mediated by DNA damage, morphological changes in the nuclei of cells and chromatin condensation were observed following DAPI and 8‐OHdG co‐staining (Figures 8 and 9). Consistent with the MTT assays, the results revealed that fentanyl and remifentanil at high concentrations have a neurotoxic effect by reducing the total cell number. Fentanyl and remifentanil were observed as + at 0.01 and 0.1, ++ 1 μg/mL, while fentanyl and remifentanil were evaluated as +++ at 10 μg/mL (*p* < 0.05).

## 4 DISCUSSION

Opioids are the drug of choice because of their pain‐relieving effect in a multitude of conditions, including post‐operative care and cancer. Fentanyl, morphine, meperidine, oxycodone, sedation, analgesia, vomiting, nausea, respiratory depression (at high doses to apnoea), bradycardia and at higher doses unconsciousness are known to cause the usual μ‐opioid receptor‐mediated central nervous system damage.^22^

In this study, we focused on the most commonly used analgesics fentanyl and remifentanil. The increase in illegal use of these opiates has led us to focus on the neurodegenerative damage of these drugs. Inflammatory responses have played a major role in the pathophysiology of various neurodegenerative diseases. In this context, neuroinflammation is characterized by the activation of resident glial cells linked to CNS immune surveillance through the release of chemokines, cytokines and other mediators that, in turn, can recruit peripheral cells. Therefore, in our study, neuroinflammation levels were determined by examining the effects on TNF, IL‐8 and IL‐10 synthesis and release, which are important inflammatory markers. In addition to our study, AChE, BChE, PON1 and total thiol levels were examined.

Neurodegenerative diseases (like Alzheimer's, multiple sclerosis, and Parkinson's) have been characterized by neuronal degeneration and death in the CNS. Neurodegeneration is mediated by inflammatory and neurotoxic mediators (IL‐1β, IL‐6, IL‐8, IL‐33, TNF‐α and chemokine ligand 2). Activated neurons, astrocytes, microglia, mast cells and T cells release these inflammatory mediators and may cause neuroinflammation and neurodegeneration. In addition, immune and inflammatory cells and inflammatory mediators from the environment cross the defective blood–brain barrier and increase neuroinflammation. This inflammation induces the release of inflammatory mediators including IL‐8 and 10 and TNF‐a. Astrocytes and microglia function as inflammatory cells and secrete lots of neuroinflammatory cytokines and chemokines in ageing.^23^ TNF‐α mediates neurotrophic/neuroprotective activity and upregulates the expression of BDNF in astrocytes in the brain.^24^, ^25^, ^26^, ^27^ The presence of systemic inflammation with increased TNF‐α production is associated with increased glial cell activation, cognitive decline and neuroinflammation.^28^ In our study, it was observed that the TNF‐α level increased 2.3 times in the fentanyl group, while this value decreased to 0.6 in the remifentanil group.

A potent anti‐inflammatory cytokine, IL‐10, has played a critical role in balancing immune responses to overcome chronic inflammatory diseases and has been thought an important anti‐inflammatory modulator of glial activation.^29^, ^30^ The production of anti‐inflammatory cytokines as IL‐10 constitutes one of the most complex mechanisms exerted by immune cells to stop excessive inflammation, paving the way for numerous studies focusing on understanding IL‐10 regulation by cells in the CNS.^29^, ^31^ In this context, the release of inflammatory mediators such as proinflammatory cytokines and free radicals is responsible for blood–brain barrier damage, having led to ischemia and brain oedema, although mainly produced to restore nerve tissue.^32^ Different experimental models, both in vitro and in vivo, have proven that IL‐10 in ischemic strokes is associated with neuroprotective effects, apart from being an interesting and clinically useful diagnostic tool in TBI patients.^33^, ^34^, ^35^ Other studies have reported that high IL‐10 levels are associated with severity and mortality in severe TBI.^36^

In addition, higher IL‐10 levels in CSF have been significantly associated with mortality in both paediatric and adult patients.^37^, ^38^, ^39^ In our study, IL‐10 level was investigated to determine the neuroinflammation caused by fentanyl and remifentanil drugs on neuron cells. As a result, it was observed that fentanyl and remifentanil applied at different concentrations increased IL‐10 levels. Almost similar levels of up‐regulation were observed in the two drug groups (1.68 and 1.65, respectively). According to these results, it was determined that neuroinflammation increased. The results explain the mechanism of the decrease in the viability level observed in the MTT cell viability test results. These results also confirm the results of the analysis regarding the cellular antioxidant system and the enzyme activity involved in the cholinergic system (cholinesterase enzymes) associated with Alzheimer's disease, which is one of the neurodegenerative diseases.

Two types of cholinesterase enzymes, AChE and butyrylcholinesterase (BChE), responsible for the hydrolysis of AChE, are found in the central nervous system (CNS).^40^ BChE, which plays a key role in the regulation of AChE activity, is known to have less hydrolytic activity than AChE.^41^, ^42^, ^43^ In the cholinergic hypothesis, inhibition of enzymes is important due to the reduction of the level of ACh hydrolysed by cholinesterase enzymes in the synaptic space. Therefore, enzyme inhibitors are used to prevent and control this decrease in ACh in the treatment of Alzheimer's disease.^44^ Intense loss of neurons in brain regions causes cholinergic disorders. As a result, it is stated in the literature that choline acetyltransferase (ChAT) and acetylcholinesterase (AChE) activity and acetylcholine (ACh) release may decrease.^45^

In this study, fentanyl and remifentanil drugs applied at different doses to neuron cell culture did not show any effect against the AChE enzyme compared to the control group but showed an inhibitory effect against BChE.^46^ These drugs may have shown an inhibitory effect against BChE, either directly or indirectly. Indirectly, these drugs may cause damage to cholinesterase metabolism with their neurotoxic effect, resulting in a decrease in the amount of neurotransmitter acetylcholine released. With this decrease, a decrease in the activity of BChE rather than AChE is expected with the decrease of acetylcholine in the environment and the ChAT activity that synthesizes it. Because acetylcholine as a substrate is more specific to AChE. If the neurotoxicity that caused the decrease in acetylcholine release had increased more, a decrease in the activity of AChE, another cholinesterase, would have been expected.

Paraoxonase (PON1) is an HDL‐related esterase with antiatherogenic properties as it protects lipoproteins and arterial cells against oxidation by hydrolysing lipid peroxides such as cholesteryl esters and phospholipids.^47^ PON1 also inhibits the accumulation of oxidized phospholipids formed by peroxynitrite by hydrolysing phosphatidylcholine (PC) isoprostanes and PC core aldehydes. PON1 plays an antioxidant role against oxidative stress. Thus, it plays an active role in the prevention of many diseases, especially atherosclerosis.^48^, ^49^, ^50^

In the study, fentanyl and remifentanil drugs applied at different doses caused inhibition of PON 1 activity in neuron cell culture when compared to controls. At these doses, remifentanil has a higher inhibitory effect on PON 1 than the other drug. The fact that fentanyl and remifentanil drugs administered at different doses cause a decrease in PON 1 activity may be a harbinger of an increase in oxidative stress in the cell. This increased oxidative stress may result in increased damage to cells. MTT results also support this claim. Due to the high decrease in PON 1 activity in cell culture treated with remifentanil, it can be expected that this drug has a higher neurotoxic effect than the other drug.

Due to enzymatic and non‐enzymatic cellular antioxidant defence systems, the development of endogenous oxidative stress‐related disease reduced the severity of oxidative damage to brain tissue. In a study, it was stated that enzymatic antioxidants such as glutathione reductase (GR), superoxide dismutase (SOD), glutathione peroxidase (GPx), and catalase (CAT) and non‐enzymatic biomarkers (GSH and total thiols) levels increased to reduce oxidative stress in the brain.^51^, ^52^ It has been shown that thiols, which are of great importance among antioxidants, play important roles in detoxification, enzymatic reactions, apoptosis and antioxidant defence systems in the body. They contain protein sulfhydryl groups, protein thiols in plasma and a protein mixture consisting of cysteine, cysteinyl glycine, GSH, and homocysteine and disulfides.^53^, ^54^ Many studies have shown that changes in thiol status and thiol/disulfide homeostasis can occur in various diseases such as cancer, respiratory, digestive and metabolic diseases.^55^ Therefore, thiol levels could regulate the metabolism / improve knowledge of the medical drug that can prevent oxidative stress‐related diseases and will provide an important contribution to the treatment.

In the study, when compared to controls, remifentanil applied at different doses increased the total thiol level in neuron cell culture, while fentanyl did not change. One of the main reasons for this increase is the increase in the amount of thiol in the cell by activating the enzymatic (CAT, GPx and GR), and non‐enzymatic antioxidant defence system against oxidative stress caused by neurotoxicity caused by drugs. As stated in the literature information given above, an increase in the amount of total thiol is expected to reduce the oxidative stress that increases with neurotoxicity. As indicated in the PON 1 activity results, oxidative stress due to oxidation of lipids was likely to be higher in the use of remifentanil. An increase in the total thiol level is expected to reduce the neurotoxicity that we think may occur due to the remifentanil administered in this study. When these results are evaluated, the use of fentanyl has fewer side effects than other drugs.

## 5 CONCLUSIONS

Based on our findings, we concluded that fentanyl and remifentanil group medications cause neuron cell death via causing inflammation as well as total thiol, BChE, PON1 and AChE levels to rise. Interestingly, remifentanil was found to have a stronger neuroinflammatory effect.

## AUTHOR CONTRIBUTIONS

**Ali Taghizadehghalehjoughi:** Conceptualization (lead); data curation (equal); formal analysis (equal); writing – original draft (equal). **Muhammet Emin Naldan:** Data curation (equal); formal analysis (equal); writing – original draft (equal). **Yesim Yeni:** Conceptualization (equal); formal analysis (equal); writing – original draft (equal). **Sidika Genc:** Conceptualization (equal); investigation (equal); methodology (equal); writing – original draft (equal). **Ahmet Hacimuftuoglu:** Investigation (equal); methodology (equal); writing – original draft (equal). **Mesut Isik:** Data curation (equal); formal analysis (equal); methodology (equal); writing – original draft (equal). **Adem Necip:** Investigation (equal); methodology (equal); writing – original draft (equal). **İsmail Bolat:** Formal analysis (equal); methodology (equal); writing – original draft (equal). **Serkan Yildirim:** Investigation (equal); methodology (equal); writing – original draft (equal). **Sukru Beydemir:** Methodology (equal); writing – original draft (equal). **Mahmut Baykan:** Investigation (equal); methodology (equal); writing – original draft (equal).

## CONFLICT OF INTEREST STATEMENT

There is no conflict of interest.

## Data Availability

The data are available on request.
